# Targeted Integration of a Super-Exon into the *CFTR* Locus Leads to Functional Correction of a Cystic Fibrosis Cell Line Model

**DOI:** 10.1371/journal.pone.0161072

**Published:** 2016-08-15

**Authors:** Christien Bednarski, Katja Tomczak, Beate vom Hövel, Wolf-Michael Weber, Toni Cathomen

**Affiliations:** 1 Institute for Cell and Gene Therapy, Medical Center–University of Freiburg, Freiburg, Germany; 2 Center for Chronic Immunodeficiency, Medical Center–University of Freiburg, Freiburg, Germany; 3 Institute of Animal Physiology, Westphalian Wilhelms-University, Muenster, Germany; 4 Faculty of Medicine, University of Freiburg, Freiburg, Germany; Imperial College London, UNITED KINGDOM

## Abstract

*In vitro* disease models have enabled insights into the pathophysiology of human disease as well as the functional evaluation of new therapies, such as novel genome engineering strategies. In the context of cystic fibrosis (CF), various cellular disease models have been established in recent years, including organoids based on induced pluripotent stem cell technology that allowed for functional readouts of CFTR activity. Yet, many of these *in vitro* CF models require complex and expensive culturing protocols that are difficult to implement and may not be amenable for high throughput screens. Here, we show that a simple cellular CF disease model based on the bronchial epithelial *ΔF508* cell line CFBE41o- can be used to validate functional CFTR correction. We used an engineered nuclease to target the integration of a super-exon, encompassing the sequences of *CFTR* exons 11 to 27, into exon 11 and re-activated endogenous *CFTR* expression by treating CFBE41o- cells with a demethylating agent. We demonstrate that the integration of this super-exon resulted in expression of a corrected mRNA from the endogenous *CFTR* promoter and used short-circuit current measurements in Ussing chambers to corroborate restored ion transport of the repaired CFTR channels. In conclusion, this study proves that the targeted integration of a large super-exon in *CFTR* exon 11 leads to functional correction of CFTR, suggesting that this strategy can be used to functionally correct all *CFTR* mutations located downstream of the 5’ end of exon 11.

## Introduction

Cystic Fibrosis (CF) is a lethal autosomal recessive inherited disorder with an approximate prevalence of 1 in 2,500 newborns among the Caucasian population. The cystic fibrosis transmembrane conductance regulator (CFTR) was linked to CF pathology right after its identification in 1989 [1–3]. CFTR is a member of the ABC transporter family and located in the membrane of many secretory epithelia found throughout the body. CFTR functions as a chloride channel, mediates conductance of ions across the membrane and is therefore important for the maintenance of ion and liquid homeostasis of the epithelia throughout the body [4,5]. Mutations in the gene encoding the CFTR channel result in impaired epithelial ion and water transport, the consequences are dysfunctional glands, thickened mucus, and eventually malfunction of the affected organs. The primary cause of mortality in CF patients is the profound bacterial infection of the conducting airways, which leads to progressive lung disease and ultimate respiratory failure. A deletion of three base pairs in exon 11 (according to nomenclature proposed by the Human Genome Variation Society, http://varnomen.hgvs.org/) of the *CFTR* gene (*ΔF508* mutation) contributes to ∼70% of all CF cases worldwide [6]. This loss of phenylalanine at position 508 results in incomplete processing and subsequent degradation of the immature CFTR protein [7].

Current treatment options for CF patients are based on pharmacological therapies and small compound correctors that try to manage and control CF symptoms, such as malnutrition, intestinal and airway blockages, and chronic bacterial infections. Many efforts have been made to develop a sustainable gene therapy based on the transfer of a wild-type copy of the *CFTR* gene to the lung [8,9] with recent success in a multi-dose trial [10]. Other promising approaches include genome editing using designer nucleases that enable the correction of specific mutations as well as the targeted insertion of foreign DNA sequences at desired genomic loci by harnessing the homology directed repair (HDR) pathway of the cell [11,12]. Since the generation of the first zinc-finger nucleases (ZFNs) and more recent developments of alternative genome targeting tools, such as transcription activator-like effector nucleases (TALENs) and CRISPR/Cas based RNA-guided nucleases (RGNs), many gene editing studies in human cells–and cells of many model organisms–have been carried out [13–15].

Considerable efforts have been invested in generating CF *in vitro* model systems to study the molecular and cellular pathophysiology of the disease on the one hand and to develop new genetic and pharmacological drugs for potential therapeutic approaches on the other hand. The spectrum of available cellular models ranges from transformed or primary cells originating from airway, pancreas or intestine epithelia [16,17] to rather complex intestinal stem cells [18].

The goal of this study was the development of a targeted genome engineering approach that allows for the genetic correction of the majority of described CF causing mutations [19,20], and to employ a simple cell line model to functionally validate this gene editing approach. The patient derived CFBE41o- cells are homozygous for the *∆F508* mutation [21,22] and have been used for drug screening in several studies [23]. Because endogenous *CFTR*-*∆F508* expression in this cell line is low, CFBE41o- cells were frequently complemented with exogenous *CFTR-∆F508* expression cassettes in order to screen for specific compounds to functionally rescue the ∆F508 mutation [24,25]. Here, we show that we could reestablish endogenous *CFTR* expression in the CFBE41o- cell line after restoration of the *ΔF508 CFTR* locus using a ZFN-based gene editing approach. We provide proof that the targeted integration of a super-exon into exon 11 reinstated expression of functional CFTR that in turn corrected transepithelial characteristics of these cells. In conclusion, our results demonstrate that a super-exon strategy can be applied to correct the majority of *CFTR* mutations and that a simple cell line model can be employed to functionally validate such gene editing approaches.

## Material and Methods

### Designer nucleases and donor construct

A plasmid encoding CFTR-ZF binding domains was kindly provided by J. Keith Joung (Massachusetts General Hospital) [26]. The coding sequence was cloned into an pRK5 expression vector, that carries heterodimeric *Fok*I variants (EA-QV) [27]. The donor construct was generated using standard cloning procedures. Homology arm left (HAL) spans 690 bp of intron 10 sequence, while homology arm right (HAR) consists of 840 bp, including exon 11 and parts of intron 11. The first 30 bp of donor exon 11 contain silent mutations to avoid ZFN cleavage of the donor and to facilitate genetic detection (sequence tag). The homology arms flank the partial *CFTR* cDNA encompassing exons 11–27 and a puromycin selection gene driven by a PGK promoter. The relevant sequence of the donor is given in S1 File. Complete sequence information can be obtained upon request.

### Cell culture

Bronchial epithelial cells CFBE41o- and 16HBE14o- were obtained from Dieter Gruenert (UCSF) and were cultured at 37°C in 5% CO_2_ in Minimal Essential Medium, supplemented with 10% FCS, 1% penicillin/streptomycin, 1% sodium pyruvate.

### Gene editing and T7EI assay

ZFN activity was determined using the T7 endonuclease I (T7EI; New England Biolabs) assay [28]. Briefly, ZFN expression plasmids were transfected (Lipofectamine 2000, Life Technologies) into CFBE41o- cells and harvested upon 48 hours. Genomic DNA was extracted with QIAquick PCR Purification Kit (Qiagen), the locus was PCR amplified (primer pair T1/T2, S1 Table), amplicon was denatured and subjected to digestion with T7EI. Mismatch recognition mediated cleavage was evaluated by 2% agarose gel electrophoresis and quantified with QuantityOne v.4.6.9 software (Biorad), as described in Mussolino *et al*. [29]. To calculate the cleavage activity, we used the formula: mutation frequency in (%) = 100 × (1 − (1 − fraction cleaved)^1/2^), where the fraction cleaved is the total relative density of the cleavage bands divided by the sum of the relative density of the cleavage bands and uncut bands. Background of mock treated samples was subtracted from all samples.

For gene targeting, 10e6 CFBE41o- cells were transfected with different ZFN expression vector to donor ratios (total of 5 μg DNA) with Nucleofector II (Lonza), using cell line Nucleofector Kit V and program A20. For pre-selection analysis, genomic DNA was extracted after 5 days with QIAamp Blood Mini Kit (Qiagen), followed by genotyping via an inside-out PCR strategy (S1 Table). For post-selection analysis, cells were cultured for an additional 4 days, subjected to selection with 1 μg/ml puromycin (Roth, Germany), expanded for 14 days, before single cell clones were obtained through limiting dilution. Genomic DNA was extracted with DirectPCR Lysis Reagent (Peqlab) and genotyped with primers listed in S1 Table.

### Immunoblotting

Western blotting was performed as described earlier [30]. Briefly, ZFNs were detected with an anti-HA tag antibody (1:1,000; Novus Biologicals), while β-actin was detected as an internal control with anti-β-actin antibody (1:1,000; Cell Signaling). Proteins were visualized with HRP-conjugated goat anti-rabbit antibody (1:10,000; Dianova) using chemiluminescence (SuperSignal West Pico; Thermo Fisher Scientific).

### RNA expression analysis

CFBE41o- growth media was supplemented with 5 μM of the demethylation agent 5-Aza-2’-deoxycytidine (Sigma) for four days to enhance expression from the *CFTR* locus. Total RNA was extracted using TRIzol (Life Technologies) phase separation procedure and subsequent isopropanol precipitation. *CFTR* cDNA was synthesized with QuantiTect Reverse Transcription Kit (Qiagen) using 500 ng of total RNA, and used for transcript analysis (S1 Table).

### Methylation analysis via bisulfite sequencing

Genomic DNA of CFBE41o- cells, 16HBE14o- cells and corrected cell clones was extracted with Qiagen Blood Mini kit, and 500 ng of genomic DNA was subjected to bisulfite treatment using EpiMark bisulfite conversion kit (New England Biolabs) according to the manufacturer’s instructions. The *CFTR* promoter region was amplified using primers specific for the converted sequence (B1/B2, S1 Table). For PCR amplification EpiMark Hot Start *Taq* DNA Polymerase was used (40 cycles, annealing temperature of 55°C). PCR products were purified from agarose gel, subcloned using the CloneJET PCR cloning kit (Thermo Fisher Scientific), and sequenced using primer pJET1.2/FW (Thermo Fisher Scientific). Methylation analysis was performed with ClustalW (http://www.genome.jp/tools/clustalw/), Quma (http://quma.cdb.riken.jp/) and Bioconverter (http://biq-analyzer.bioinf.mpi-inf.mpg.de/tools/BiConverter/index.php) web based software.

### Transepithelial measurements

CFBE41o- cells were cultivated for 14–18 days on filter supports (Costar, Corning) before measurements in modified Ussing chambers (designed by Prof. Dr. Willy Van Driessche; KU Leuven, Belgium) were performed. The used Ag/AgCl electrodes were connected to the Ringer solution (130 mM NaCl, 5 mM KCl, 2 mM MgCl_2_, 1 mM CaCl_2_, 5 mM glucose, 10 mM HEPES pH 7.3). Transepithelial potential (V_t_) was clamped to 0 mV with a low-noise voltage clamp. Transepithelial short-circuit current (I_sc_) was continuously recorded (ImpDsp 1.4; KU Leuven). All values were normalized to 1 cm^2^. Upon adapting the cells to Ringer solution, an activating cocktail containing the membrane permeable cAMP analogue 8-[4-chlorophenylthio (CTP)]-cAMP (100 mM, Biolog, Bremen, Germany) and IBMX (1 mM, AppliChem GmbH, Darmstadt, Germany) was applied to the basolateral side of the monolayer. CFTR current was inhibited using the specific CFTR blocker CFTRinh172 (20 μM, Tocris Bioscience, Bristol, UK).

### Statistics

Data are shown as the arithmetic mean ± SEM (standard error of the mean). “n” is the number of replications. Where applicable, statistical evaluations were performed with the Student’s *t*-test for independent, unpaired samples. Statistically significant differences (p ≤ 0.01) are marked with “*”. All statistical tests were performed with Origin 7.0 (OriginLab Corporation, Northampton, USA).

## Results

### ZFN-mediated cleavage of *CFTR* exon 11

**Fig 1:**
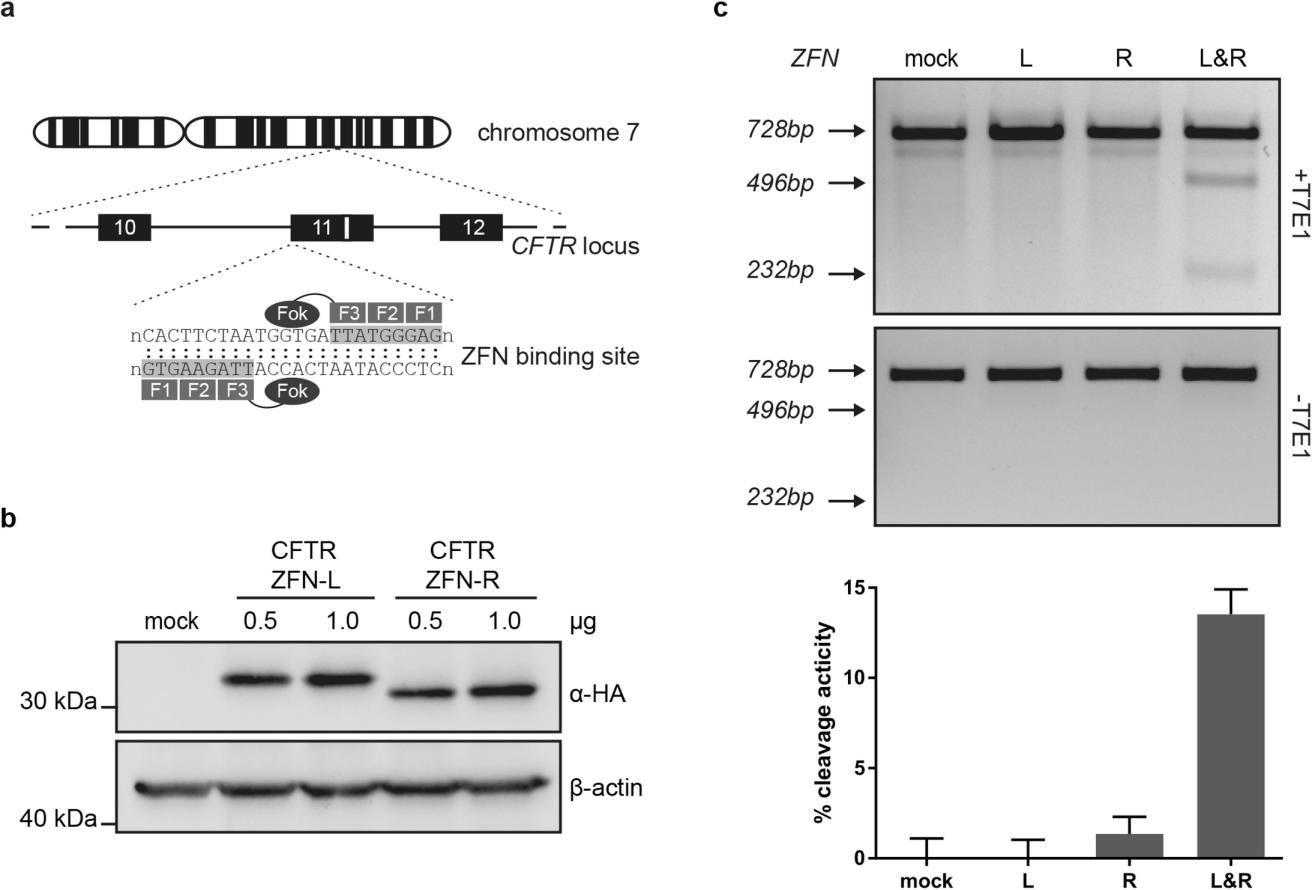
Functionality of *CFTR* specific ZFNs in human CFBE41o- bronchial epithelial cells. (a) Schematic of ZFN binding site in *CFTR* exon 11. *ΔF508* mutation is depicted as white box. The ZFN binds to two target half-sites separated by a 6 bp spacer. (b) ZFN expression levels. CFBE41o- cells were transfected with indicated amounts of ZFN expression vectors and harvested to determine ZFN expression levels by immunoblotting using HA tag specific antibodies. ß-actin was included as a loading control. (c) ZFN cleavage activity. CFBE41o- cells were transfected with 0.5 μg of ZFN expression plasmid, either left (L) or right (R) subunit alone, or in combination (L&R). A 728 bp PCR amplicon encompassing the ZFN cleavage site was subjected to digestion with the mismatch-sensitive T7 endonuclease I (T7EI). Expected positions of the cleavage products are indicated, an asterisk indicates an unspecific cleavage product. Representative gels of a total of four experiments (n = 4) are shown on top. Average cleavage activity is shown on the bottom, where error bars indicate standard error of the mean (SEM).

In order to introduce a therapeutic *CFTR* cassette in exon 11 of the *CFTR* locus, ZFNs designed to target an 18 bp stretch at the 5’ end of exon 11 were used to insert a DNA double strand break (DSB) located 130 bp upstream of the *ΔF508* mutation (Fig 1A). To examine the expression of the CFTR-specific ZFNs, two concentrations of ZFN expression plasmids were transfected into target cell line CFBE41o-, which is homozygous for the *ΔF508* mutation. Western blot analysis detecting HA-tagged ZFNs confirmed protein expression 48 hours post transfection (Fig 1B). Note, the two ZFN subunits migrate differently in SDS-PAGE due to differently charged *Fok*I domains [27,30]. Assessment of the endogenous cleavage activity of the nuclease using the mismatch-sensitive T7 endonuclease I (T7EI) confirmed sequence-specific cleavage of approximately 14% of *CFTR* alleles (Fig 1C).

### Targeted insertion of a partial *CFTR* cDNA into exon 11

**Fig 2:**
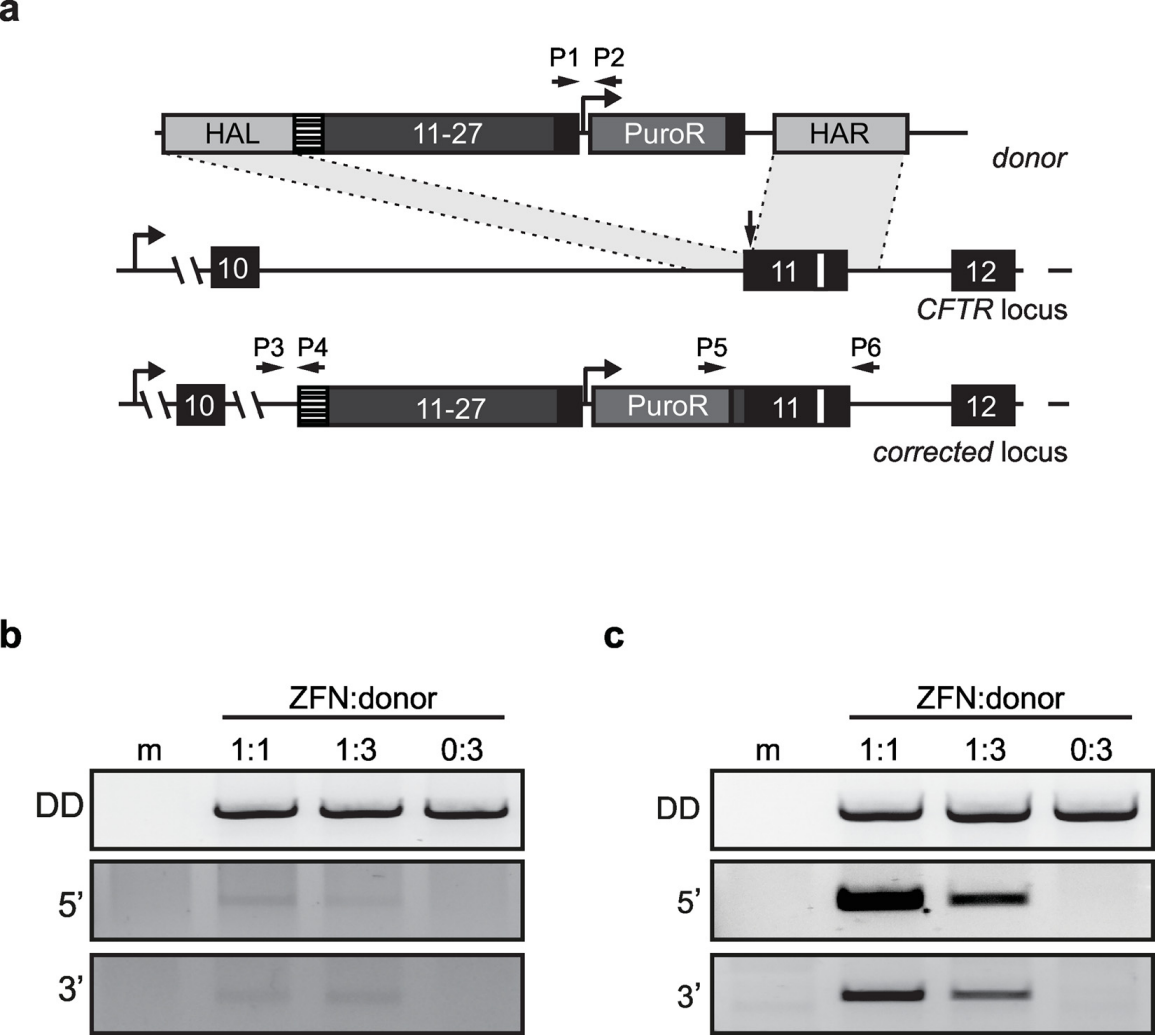
Targeted insertion of a therapeutic donor in *CFTR* exon 11. (a) Schematic. ZFN mediated cleavage of the target site stimulates homologous recombination (HR) with the donor template. The donor carries a *CFTR* cDNA comprising exons 11–27 and a puromycin selection cassette (PuroR), flanked by homology arms (HAL, homology arm left; HAR, homology arm right) of 750–850 bp. Positions of primer binding sites are indicated, *ΔF508* mutation is depicted as white box, vertical arrow indicates ZFN binding site and hatched box represents 30 bp modified region to prevent ZFN binding to the donor (also referred to as sequence tag). (b) Pre-selection analysis. CFBE41o- cells were transfected with different ZFN:donor ratios (0:3 indicates absence of ZFN expression vectors). The polyclonal cell population was subjected to PCR-based genotyping 3 days post-transfection with the indicated primers: Primer pairs P1/P2 for donor detection (DD), P3/P4 for detection of 5’ junction (5’), P5/P6 for detection of 3’ junction (3’). (c) Post-selection analysis. The transfected cells were subjected to puromycin selection and the polyclonal cell population assessed by PCR-based genotyping as in (b).

To functionally correct the *ΔF508* mutation in CFBE41o- cells, we designed a therapeutic donor construct which contained exons 11–27 of the *CFTR* gene, here designated as super-exon (Fig 2A). This design will allow us to correct all *CFTR* mutations located downstream of exon 11 and can be combined with antibiotic selection of targeted cell clones. To avoid cleavage of the donor DNA by co-expressed ZFNs, silent mutations were introduced into the first 30 bp of donor exon 11. This modification also facilitated screening for correctly integrated donor sequences via PCR genotyping and was therefore designated as “sequence tag”. For ZFN-mediated gene targeting, CFBE41o- cells were transfected with different ZFN:donor ratios (Fig 2B) and assessed for site-specific integration. As expected, signals for correct targeting of the *CFTR* locus was detected only in CFBE41o- cells expressing ZFNs. After transfection, cells were treated with puromycin to enrich for cells with a correctly targeted genome (Fig 2C). As expected, the presence of donor DNA was confirmed in all samples treated with ZFNs before and after antibiotic selection. However, we also detected the donor sequence after selection in samples not transfected with ZFN expression vectors, which may be due to randomly integrated donor.

### Clonal analysis confirms targeted correction of the *CFTR* locus

**Fig 3:**
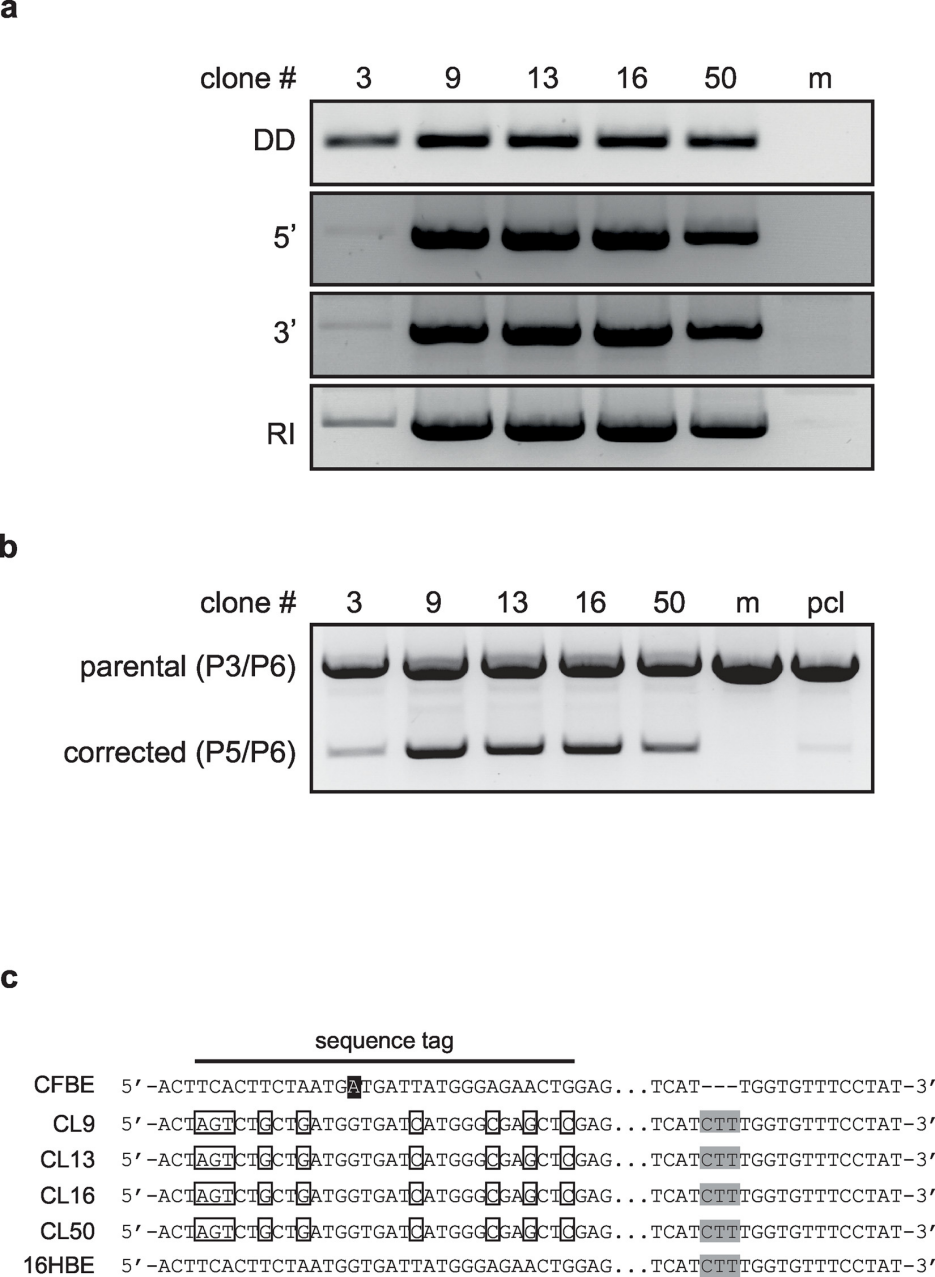
Genotype analysis of targeted CFBE41o- clones. (a) PCR-based genotyping. Genetic analysis of the corrected CFBE41o- clones was carried out as described for Fig 2. DD, donor detection; 5’, 5’ junction; 3’, 3’ junction; RI, random integration. RI was detected with primers R1/R2, amplifying parts of the donor backbone (see S1 Table) (b) Allelic discrimination. A three primer based PCR approach was employed to discriminate between parental allele (amplification by primer pair P3/P6) and targeted allele (P5/P6). Positions of PCR primers are shown in Fig 2A; pcl, polyclonal population. (c) Sequence of restored alleles. The CTT triplet is highlighted in gray and the sequence tag at the 5’ end of exon 11 is indicated. The single nucleotide polymorphism (SNP; G A, rs213950) present in CFBE41o- cells is highlighted in black. The sequence of 16HBE cells is indicated as a reference.

Single cell-derived clones were obtained by limited dilution of the polyclonal CFBE41o- population. A total of 138 clones were screened for targeted integration of the donor by PCR genotyping. Five out of 48 clones from CFBE41o- cells transfected with a 1:1 ZFN:donor ratio showed successful targeted integration (Fig 3A), while no clones were identified when using a ZFN:donor ratio of 1:3. This coincides with the polyclonal PCR targeting profile seen in Fig 2B. These results indicate a targeting frequency of ~10% when an optimal ZFN:donor ratio was applied. Next, we analyzed allelic distribution of the targeted clones with a PCR-based allelic discrimination strategy. All targeted clones displayed monoallelic correction that is all cells contain a *ΔF508* allele as well as a targeted *CFTR* allele (Fig 3B). Of note, clone #3 was excluded from all subsequent analyses because of genetic instability. Accurate targeted insertion was verified by sequence analysis, confirming restoration of the CTT triplet and the presence of the inserted sequence tag, i.e. the silent mutations originally present on the donor plasmid (Fig 3C and S1 Fig).

### Genotypic corrected CFBE41o- cells express *CFTR* mRNA

**Fig 4:**
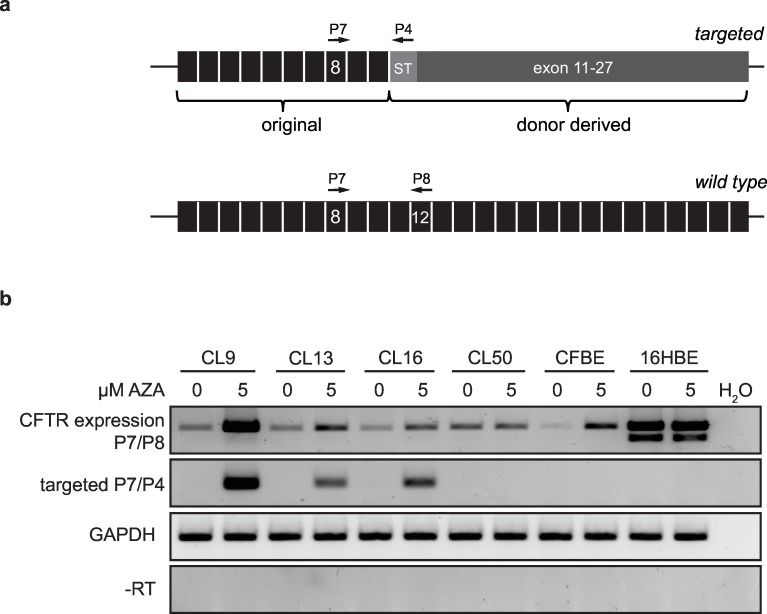
Gene edited clones express corrected *CFTR* mRNA. (a) Allele-specific RT-PCR strategy. Primer pairs P7/P8 were used to amplify both edited and unedited alleles of *CFTR* mRNA while combination P7/P4 amplified only the corrected mRNA. ST, sequence tag. (b) *CFTR* mRNA expression levels. The effect of 5-Aza-2'-deoxycytidine (AZA) on *CFTR* expression in CFBE41o- cells and the gene edited clones was determined semi-quantitatively by RT-PCR using either primer pairs P7/P8 or P7/P4. The detection of *CFTR* mRNA in 16HBE14o- cells and *GAPDH* mRNA served as positive controls, samples without reverse transcriptase (-RT) as a negative control.

Next, we asked if a stable mRNA transcript is expressed from the corrected *CFTR* alleles. In contrast to wild-type 16HBE cells, *CFTR* mRNA is expressed at very low levels in CFBE41o- cells (S2B Fig). We hypothesized that the low mRNA levels were due to epigenetic silencing of the *CFTR* locus in this cell line. However, this silencing is not linked to the ∆F508 mutation, as primary CF patient cells express high levels of *CFTR* mRNA [31]. Bisulfite sequencing of a 360 bp stretch of the *CFTR* promoter in CFBE41o- cells and the gene edited CFBE41o- clones confirmed the presence of methylated CpG islands (S2D Fig), a characteristic of epigenetic silencing. Treatment of cells with 5-aza-2'-deoxycytidine (AZA), an inhibitor of DNA methyltransferase, resulted in reactivation of the *CFTR* locus and well detectable *CFTR* mRNA levels in parental CFBE41o- cells as well as in the gene edited clones #9, #13 and #16 but not # 50 (Fig 4, S2B and S2C Fig). An allele-specific RT-PCR (Fig 4A) was established and used to demonstrate that ZFN-mediated gene correction resulted in expression of a chimeric CFTR mRNA in clones #9, #13 and #16 but not # 50, which might be due to differential silencing after gene-editing in that clone (Fig 4B). This provided proof that the targeted insertion of super-exon 11–27 was successful. The smaller PCR product observed in samples derived from 16HBE cells could represent a previously described splice product, resulting from exon 10 skipping [32,33].

### Gene editing in CFBE41o- clones rescued transepithelial characteristics

**Fig 5:**
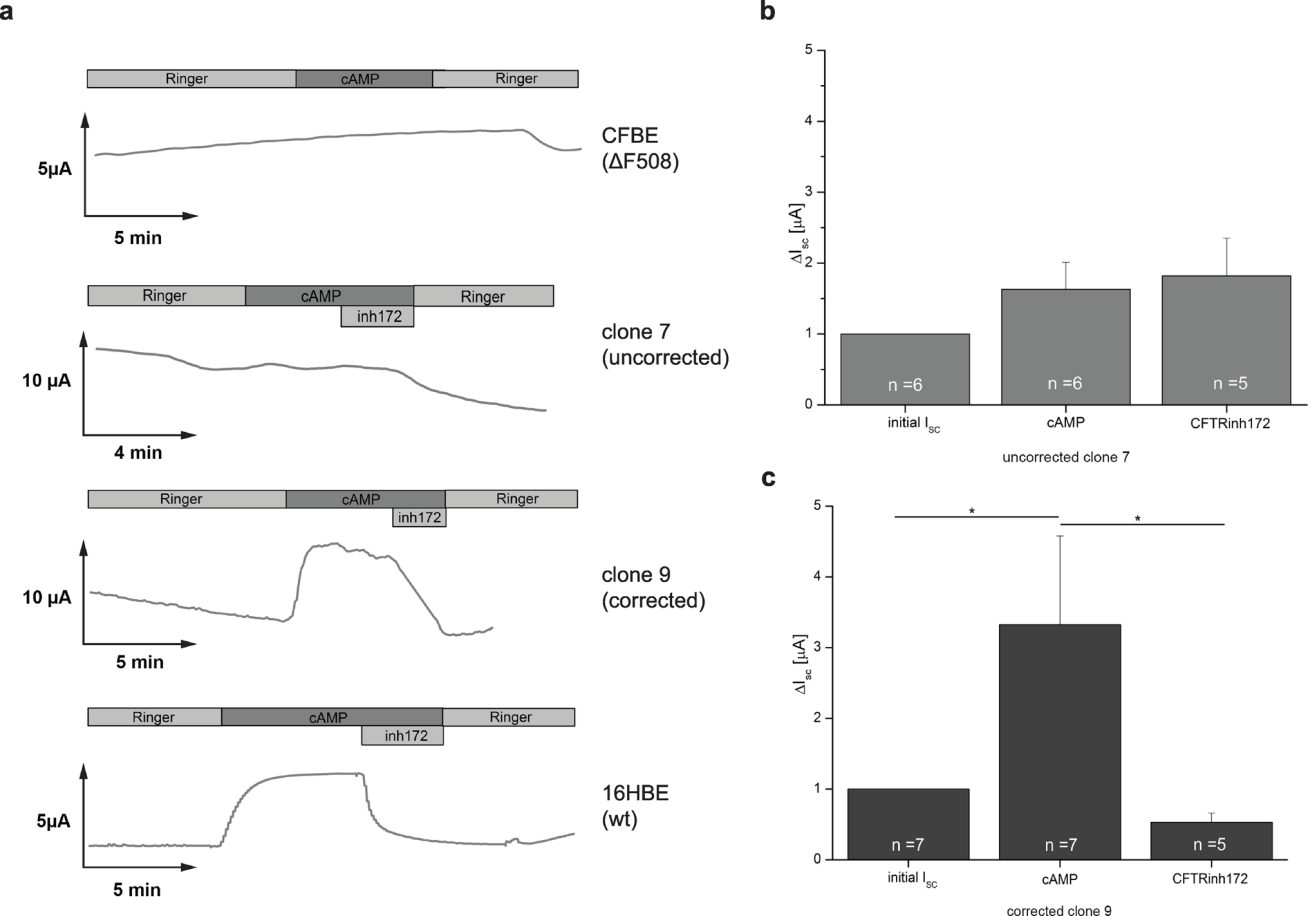
Functional restoration of CFTR channel activity. (a) CFTR activity. Representative measurements of short-circuit current (I_SC_) upon application of the cAMP activation cocktail of parental CFBE41o- cells, uncorrected clone #7, corrected clone #9, and wild-type 16HBE cells in Ussing chamber experiments. (b, c) Statistical analysis. Transepithelial measurements of clone #7 (b) and corrected clone #9 (c). The number of experiments is indicated. Bars represent mean ± SEM.

To verify that genetic correction restores CFTR function, we assessed CFTR specific transepithelial bioelectric characteristics. Transepithelial short-circuit currents (I_SC_) were determined in a modified Ussing chamber for corrected clone #9, a clone with a randomly integrated donor plasmid (clone #7), the parental CFBE41o- cells, and the 16HBE wild-type control cells. Upon application of the cAMP analog 8-[4-chlorophenylthio (CTP)]-cAMP/IBMX, activation of the I_SC_ in parental CFBE41o- cells was not detectable (Fig 5A), reflecting the absence of the CFTR channel on the apical membrane due to premature degradation of the ∆F508-CFTR protein. In contrast, the gene targeted clone #9 showed I_SC_ comparable to the I_SC_ of 16HBE wild-type cells (Fig 5A and 5B). To further verify that the cAMP/IBMX activated short-circuit current originates from CFTR-mediated chloride conductance, cells were treated with the specific CFTR-channel inhibitor CFTRinh172. Initial cAMP/IBMX activated I_SC_ of corrected clone #9 was effectively blocked with CFTRinh172, confirming that the measured current was a result of the restored activity of CFTR channels on the cell membrane (Fig 5A and 5B). Moreover, clone #7 did neither display specific activation nor any response to CFTRinh172, emphasizing that the functional restoration of CFTR function requires targeted integration of CFTR super-exon 11–27 (Fig 5B).

## Discussion

Small molecule drugs and gene therapy hold great promise to treat cystic fibrosis or even cure the disease in the future. Our study aimed at genetic correction and functional restoration of CFTR in a relevant cellular airway model system. The bronchial epithelial cell line CFBE41o- was derived from a CF patient carrying a homozygous *ΔF508* mutation, and hence seems a suitable *in vitro* model for evaluating small molecule compounds or gene editing approaches for their ability to restore CFTR function [34,35]. However, the low endogenous *CFTR* expression levels [21,22] prevented CFBE41o- cells from being used in functional screens for compounds to rescue the ∆F508 mutation without prior complementation by exogenous *∆F508-CFTR* expression [24,25].

Targeted gene editing represents a promising option for permanent correction of genetic diseases ever since the first ZFN mediated gene targeting studies designer nucleases have been put forward as DNA modifier tools with high clinical potential [36,37]. A major advantage of locus-specific gene correction over conventional gene therapy is that physiological regulation of gene expression by the endogenous promoter is retained [38,39]. Although it is not yet clear what the ultimate cellular target will be, nuclease-mediated gene editing at the *CFTR* locus has seen a surge of interest by the research community [18,40,41].

Previously, it has been shown in a proof-of-concept study that a super-exon strategy with ZFNs is feasible in a hemophilia mouse model [42]. Our approach aimed at targeted insertion of a therapeutic super-exon encompassing exons 11 to 27 into the 5’ end of exon 11, which would allow for genetic correction of all mutations downstream of the insertion site. Due to the complex transcriptional regulation of the *CFTR* locus [43,44], one of the key questions in our study was whether our super-exon integration strategy was feasible and whether it would restore normal *CFTR* expression. To this end we had to restore CFTR mRNA expression in CFBE41o- cells. Analysis of the *CFTR* promoter revealed methylation of distinct CpG sites, a hallmark of epigenetic silencing. This epigenetic remodeling could have been induced by long-term culturing of the cells or by cellular stress due to unfolded protein response triggered by immature CFTR protein [45,46]. Upon treatment of the CFBE41o- cells with AZA, CFTR mRNA expression was rescued. This observation is in line with previous studies showing elevated CFTR mRNA levels after treatment of cells with chromatin modifying compounds like sodium butyrate or histone deacetylase inhibitors [47].

Our ZFN based insertion strategy resulted in monoallelic correction of the *ΔF508* locus, which corresponds to a heterozygous *CFTR*^*wt*^*/CFTR*^*ΔF508*^ locus. This result is comparable to the outcome of a recent CRISPR/Cas based gene editing approach, in which mainly single allele targeting was observed [40]. By applying a transepithelial conductance analysis we proved that monoallelic correction of the locus is sufficient to restore CFTR dependent Cl- conductance.

On the other hand, we observed a substantial amount of random integration of donor DNA, even in the presence of ZFNs, which we did not observe to the same extent before in other human cell lines or primary human cells [48,49]. We speculate that this high frequency of illegitimate recombination is the result of high non-homologous end-joining (NHEJ) activity [50] in CFBE41o- cells, or a highly unstable genome as shown for other transformed cell lines [51]. Also, we cannot exclude that low specificity of the used ZFNs contributed to the high frequency of improper recombination [52]. Alternative designer nucleases with proven high specificity, such as TALENs or second generation CRISPR/Cas systems [53–57], can be used instead. In any case, before clinical translation designer nuclease associated off-target activity as well as illegitimate recombination of the donor DNA must be closely determined and monitored [58].

In conclusion, we provide functional proof that targeted insertion of a super-exon gives rise to active CFTR chloride channels in a simple cellular CF *in vitro* model. Moreover, we showed that AZA is a useful *in vitro* tool to reinstate CFTR expression in CFBE cells.

## Supporting Information

S1 Fig Sequence analysis of corrected clones. The corrected alleles of clones 9, 13, 16 and 50 were PCR amplified and analyzed by sequencing. The CTT triplet at position 508 is highlighted on top of the chromatogram. Silent mutations arising from the modified donor sequence in exon 11 are highlighted with asterisks (sequence tag). The single nucleotide polymorphism (SNP; GA, rs213950) present in CFBE41o- cells is highlighted with an arrow. The sequence of 16HBE14o- cells serves as a reference.
(TIF)

S2 Fig *CFTR* expression profile of genetically engineered CFBE41o- clones. (a) Simplified illustration of *de novo* methylation. The DNA (cytosine-5)-methyltransferase (DNMT) inhibitor 5-aza-2'-deoxycytidine (AZA) blocks *de novo* methylation (orange ellipses) of DNA in the cell. (b) Effect of AZA on *CFTR* mRNA expression. CFBE41o- cells were treated with increasing AZA concentrations for four or six days to re-activate the *CFTR* promoter. (c) Effect of AZA on *CFTR* mRNA expression in corrected cells. Corrected CFBE41o- clones were treated with increasing concentrations of AZA for four days and then assessed for total *CFTR* mRNA expression and donor-derived *CFTR* mRNA expression by RT-PCR. GAPDH was used as an internal control and samples without reverse transcriptase (-RT) served as a negative control. (d) Methylation profile of genetically corrected clones. The *CFTR* core promoter region (1200 bp, red) was screened for CpG islands and assessed for methylation at 20 distinct CpG sites. The extracted genomes of corrected cell clones, parental CFBE41o- cells or wild-type 16HBE14o- cells were sodium bisulfite converted, a 360 bp region was amplified (primers B1/B2) and sequenced. Black circles represent methylated and white circles represent unmethylated CpG sites, average reads of n = 4 for each clone.
(TIF)

S1 File CFTR super-exon donor sequence. DNA sequence consists of homology arm left and right (black), CFTR exon 11–27 (red), BGH polyA (green), PGK promoter (black, underlined), puromycin (blue) and SV40 polyA (black, gray shade).
(DOCX)

S1 Table Primers used for T7EI assay, genotyping and expression analysis. (DOCX)
