# Supplementary figures and images for: Targeted Integration of a Super-Exon into the CFTR Locus Leads to Functional Correction of a Cystic Fibrosis Cell Line Model
Source: PLoS One. 2016 Aug 15;11(8):e0161072. doi: 10.1371/journal.pone.0161072 (PMC4985144; doi:10.1371/journal.pone.0161072)

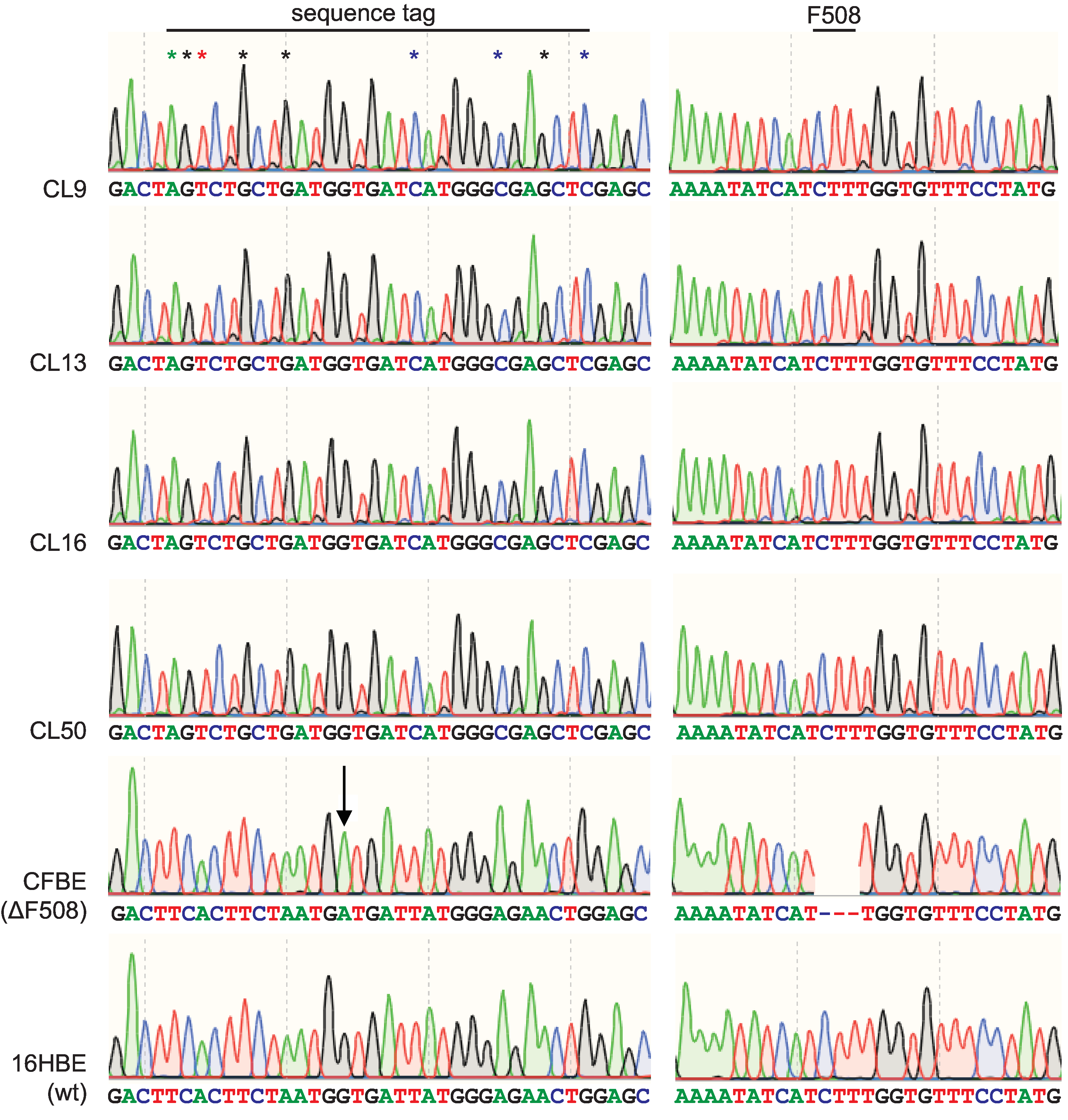

Supplement: S1 Fig — The corrected alleles of clones 9, 13, 16 and 50 were PCR amplified and analyzed by sequencing. The CTT triplet at position 508 is highlighted on top of the chromatogram. Silent mutations arising from the modified donor sequence in exon 11 are highlighted with asterisks (sequence tag). The single nucleotide polymorphism (SNP; GA, rs213950) present in CFBE41o- cells is highlighted with an arrow. The sequence of 16HBE14o- cells serves as a reference. (TIF) [file pone.0161072.s001.tif]

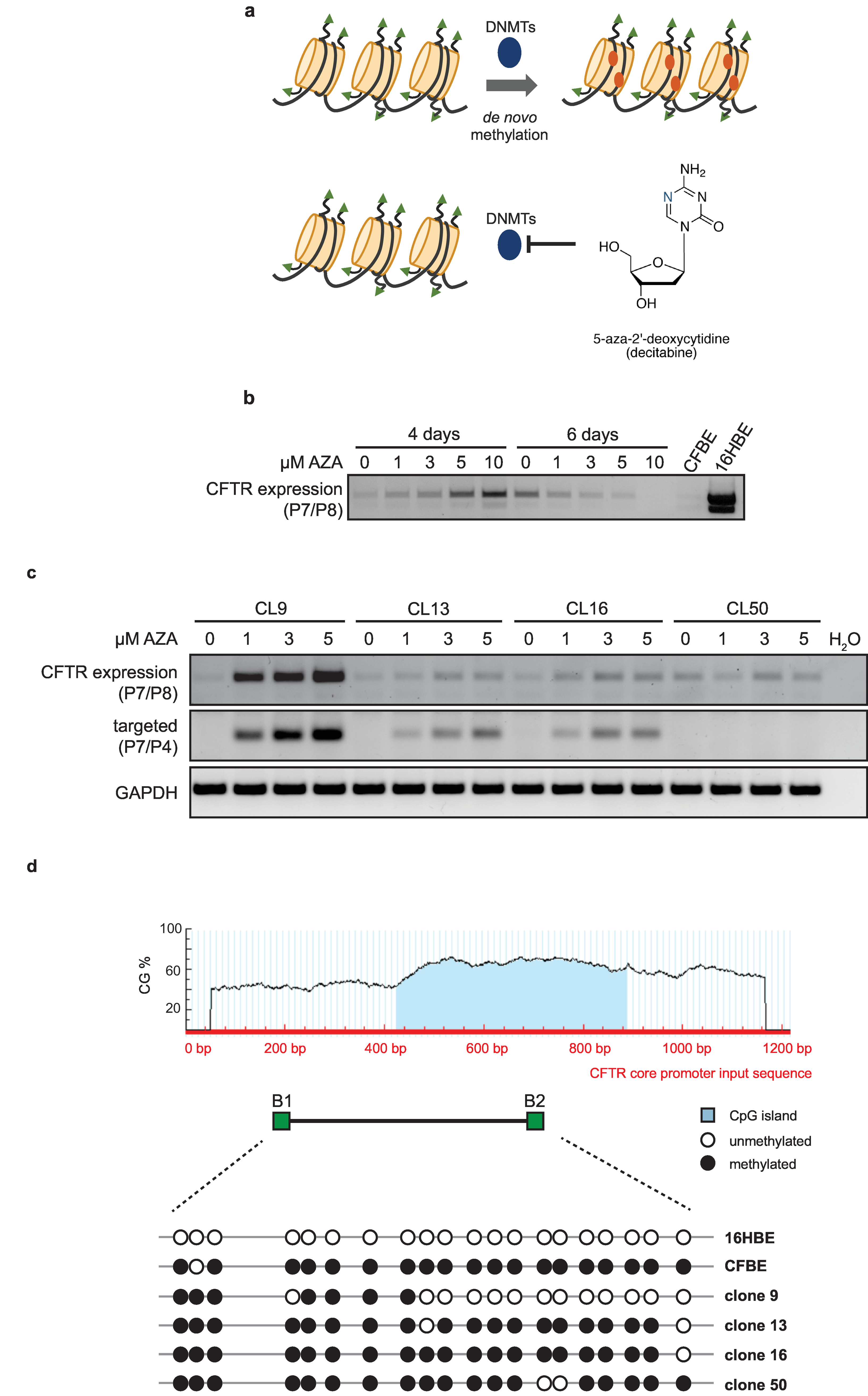

Supplement: S2 Fig — (a) Simplified illustration of de novo methylation. The DNA (cytosine-5)-methyltransferase (DNMT) inhibitor 5-aza-2'-deoxycytidine (AZA) blocks de novo methylation (orange ellipses) of DNA in the cell. (b) Effect of AZA on CFTR mRNA expression. CFBE41o- cells were treated with increasing AZA concentrations for four or six days to re-activate the CFTR promoter. (c) Effect of AZA on CFTR mRNA expression in corrected cells. Corrected CFBE41o- clones were treated with increasing concentrations of AZA for four days and then assessed for total CFTR mRNA expression and donor-derived CFTR mRNA expression by RT-PCR. GAPDH was used as an internal control and samples without reverse transcriptase (-RT) served as a negative control. (d) Methylation profile of genetically corrected clones. The CFTR core promoter region (1200 bp, red) was screened for CpG islands and assessed for methylation at 20 distinct CpG sites. The extracted genomes of corrected cell clones, parental CFBE41o- cells or wild-type 16HBE14o- cells were sodium bisulfite converted, a 360 bp region was amplified (primers B1/B2) and sequenced. Black circles represent methylated and white circles represent unmethylated CpG sites, average reads of n = 4 for each clone. (TIF) [file pone.0161072.s002.tif]
