# Supplementary material for: Targeted Integration of a Super-Exon into the CFTR Locus Leads to Functional Correction of a Cystic Fibrosis Cell Line Model
Source: PLoS One. 2016 Aug 15;11(8):e0161072. doi: 10.1371/journal.pone.0161072 (PMC4985144; doi:10.1371/journal.pone.0161072)
Supplement: S1 File — DNA sequence consists of homology arm left and right (black), CFTR exon 11–27 (red), BGH polyA (green), PGK promoter (black, underlined), puromycin (blue) and SV40 polyA (black, gray shade). (DOCX) [file pone.0161072.s003.docx]

**S1 File**

**CFTR super-exon donor sequence**

ACACTCCACACTTATACCCCATTTCCTTTGTTTGTTTATTTGGTTTTTACTTCTAACTTTTCTTATTGTCAGGACATATAACATATTTAAACTTTGTTTTTCAACTCGAATTCTGCCATTAGTTTTAATTTTTGTTCACAGTTATATAAATCTTTGTTCACTGATAGTCCTTTTGTACTATCATCTCTTAAATGACTTTATACTCCAAGAAAGGCTCATGGGAACAATATTACCTGAATATGTCTCTATTACTTAATCTGTACCTAATAATATGAAGGTAATCTACTTTGTAGGATTTCTGTGAAGATTAAATAAATTAATATAGTTAAAGCACATAGAACAGCACTCGACACAGAGTGAGCACTTGGCAACTGTTAGCTGTTACTAACCTTTCCCATTCTTCCTCCAAACCTATTCCAACTATCTGAATCATGTGCCCCTTCTCTGTGAACCTCTATCATAATACTTGTCACACTGTATTGTAATTGTCTCTTTTACTTTCCCTTGTATCTTTTGTGCATAGCAGAGTACCTGAAACAGGAAGTATTTTAAATATTTTGAATCAAATGAGTTAATAGAATCTTTACAAATAAGAATATACACTTCTGCTTAGGATGATAATTGGAGGCAAGTGAATCCTGAGCGTGATTTGATAATGACCTAATAATGATGGGTTTTATTTCCAGACTAGTCTGCTGATGGTGATCATGGGCGAGCTCGAGCCTTCAGAGGGTAAAATTAAGCACAGTGGAAGAATTTCATTCTGTTCTCAGTTTTCCTGGATTATGCCTGGCACCATTAAAGAAAATATCATCTTTGGTGTTTCCTATGATGAATATAGATACAGAAGCGTCATCAAAGCATGCCAACTAGAAGAGGACATCTCCAAGTTTGCAGAGAAAGACAATATAGTTCTTGGAGAAGGTGGAATCACACTGAGTGGAGGTCAACGAGCAAGAATTTCTTTAGCAAGAGCAGTATACAAAGATGCTGATTTGTATTTATTAGACTCTCCTTTTGGATACCTAGATGTTTTAACAGAAAAAGAAATATTTGAAAGCTGTGTCTGTAAACTGATGGCTAACAAAACTAGGATTTTGGTCACTTCTAAAATGGAACATTTAAAGAAAGCTGACAAAATATTAATTTTGCATGAAGGTAGCAGCTATTTTTATGGGACATTTTCAGAACTCCAAAATCTACAGCCAGACTTTAGCTCAAAACTCATGGGATGTGATTCTTTCGACCAATTTAGTGCAGAAAGAAGAAATTCAATCCTAACTGAGACCTTACACCGTTTCTCATTAGAAGGAGATGCTCCTGTCTCCTGGACAGAAACAAAAAAACAATCTTTTAAACAGACTGGAGAGTTTGGGGAAAAAAGGAAGAATTCTATTCTCAATCCAATCAACTCTATACGAAAATTTTCCATTGTGCAAAAGACTCCCTTACAAATGAATGGCATCGAAGAGGATTCTGATGAGCCTTTAGAGAGAAGGCTGTCCTTAGTACCAGATTCTGAGCAGGGAGAGGCGATACTGCCTCGCATCAGCGTGATCAGCACTGGCCCCACGCTTCAGGCACGAAGGAGGCAGTCTGTCCTGAACCTGATGACACACTCAGTTAACCAAGGTCAGAACATTCACCGAAAGACAACAGCATCCACACGAAAAGTGTCACTGGCCCCTCAGGCAAACTTGACTGAACTGGATATATATTCAAGAAGGTTATCTCAAGAAACTGGCTTGGAAATAAGTGAAGAAATTAACGAAGAAGACTTAAAGGAGTGCTTTTTTGATGATATGGAGAGCATACCAGCAGTGACTACATGGAACACATACCTTCGATATATTACTGTCCACAAGAGCTTAATTTTTGTGCTAATTTGGTGCTTAGTAATTTTTCTGGCAGAGGTGGCTGCTTCTTTGGTTGTGCTGTGGCTCCTTGGAAACACTCCTCTTCAAGACAAAGGGAATAGTACTCATAGTAGAAATAACAGCTATGCAGTGATTATCACCAGCACCAGTTCGTATTATGTGTTTTACATTTACGTGGGAGTAGCCGACACTTTGCTTGCTATGGGATTCTTCAGAGGTCTACCACTGGTGCATACTCTAATCACAGTGTCGAAAATTTTACACCACAAAATGTTACATTCTGTTCTTCAAGCACCTATGTCAACCCTCAACACGTTGAAAGCAGGTGGGATTCTTAATAGATTCTCCAAAGATATAGCAATTTTGGATGACCTTCTGCCTCTTACCATATTTGACTTCATCCAGTTGTTATTAATTGTGATTGGAGCTATAGCAGTTGTCGCAGTTTTACAACCCTACATCTTTGTTGCAACAGTGCCAGTGATAGTGGCTTTTATTATGTTGAGAGCATATTTCCTCCAAACCTCACAGCAACTCAAACAACTGGAATCTGAAGGCAGGAGTCCAATTTTCACTCATCTTGTTACAAGCTTAAAAGGACTATGGACACTTCGTGCCTTCGGACGGCAGCCTTACTTTGAAACTCTGTTCCACAAAGCTCTGAATTTACATACTGCCAACTGGTTCTTGTACCTGTCAACACTGCGCTGGTTCCAAATGAGAATAGAAATGATTTTTGTCATCTTCTTCATTGCTGTTACCTTCATTTCCATTTTAACAACAGGAGAAGGAGAAGGAAGAGTTGGTATTATCCTGACTTTAGCCATGAATATCATGAGTACATTGCAGTGGGCTGTAAACTCCAGCATAGATGTGGATAGCTTGATGCGATCTGTGAGCCGAGTCTTTAAGTTCATTGACATGCCAACAGAAGGTAAACCTACCAAGTCAACCAAACCATACAAGAATGGCCAACTCTCGAAAGTTATGATTATTGAGAATTCACACGTGAAGAAAGATGACATCTGGCCCTCAGGGGGCCAAATGACTGTCAAAGATCTCACAGCAAAATACACAGAAGGTGGAAATGCCATATTAGAGAACATTTCCTTCTCAATAAGTCCTGGCCAGAGGGTGGGCCTCTTGGGAAGAACTGGATCAGGGAAGAGTACTTTGTTATCAGCTTTTTTGAGACTACTGAACACTGAAGGAGAAATCCAGATCGATGGTGTGTCTTGGGATTCAATAACTTTGCAACAGTGGAGGAAAGCCTTTGGAGTGATACCACAGAAAGTATTTATTTTTTCTGGAACATTTAGAAAAAACTTGGATCCCTATGAACAGTGGAGTGATCAAGAAATATGGAAAGTTGCAGATGAGGTTGGGCTCAGATCTGTGATAGAACAGTTTCCTGGGAAGCTTGACTTTGTCCTTGTGGATGGGGGCTGTGTCCTAAGCCATGGCCACAAGCAGTTGATGTGCTTGGCTAGATCTGTTCTCAGTAAGGCGAAGATCTTGCTGCTTGATGAACCCAGTGCTCATTTGGATCCAGTAACATACCAAATAATTAGAAGAACTCTAAAACAAGCATTTGCTGATTGCACAGTAATTCTCTGTGAACACAGGATAGAAGCAATGCTGGAATGCCAACAATTTTTGGTCATAGAAGAGAACAAAGTGCGGCAGTACGATTCCATCCAGAAACTGCTGAACGAGAGGAGCCTCTTCCGGCAAGCCATCAGCCCCTCCGACAGGGTGAAGCTCTTTCCCCACCGGAACTCAAGCAAGTGCAAGTCTAAGCCCCAGATTGCTGCTCTGAAAGAGGAGACAGAAGAAGAGGTGCAAGATACAAGGCTTTAGTTAATTAAACGGCCCTATTCTATAGTGTCACCTAAATGCTAGAGCTCGCTGATCAGCCTCGACTGTGCCTTCTAGTTGCCAGCCATCTGTTGTTTGCCCCTCCCCCGTGCCTTCCTTGACCCTGGAAGGTGCCACTCCCACTGTCCTTTCCTAATAAAATGAGGAAATTGCATCGCATTGTCTGAGTAGGTGTCATTCTATTCTGGGGGGTGGGGTGGGGCAGGACAGCAAGGGGGAGGATTGGGAAGACAATAGCGTCGACGGGGTTGCGCCTTTTCCAAGGCAGCCCTGGGTTTGCGCAGGGACGCGGCTGCTCTGGGCGTGGTTCCGGGAAACGCAGCGGCGCCGACCCTGGGTCTCGCACATTCTTCACGTCCGTTCGCAGCGTCACCCGGATCTTCGCCGCTACCCTTGTGGGCCCCCCGGCGACGCTTCCTGCTCCGCCCCTAAGTCGGGAAGGTTCCTTGCGGTTCGCGGCGTGCCGGACGTGACAAACGGAAGCCGCACGTCTCACTAGTACCCTCGCAGACGGACAGCGCCAGGGAGCAATGGCAGCGCGCCGACCGCGATGGGCTGTGGCCAATAGCGGCTGCTCAGCAGGGCGCGCCGAGAGCAGCGGCCGGGAAGGGGCGGTGCGGGAGGCGGGGTGTGGGGCGGTAGTGTGGGCCCTGTTCCTGCCCGCGCGGTGTTCCGCATTCTGCAAGCCTCCGGAGCGCACGTCGGCAGTCGGCTCCCTCGTTGACCGAATCACCGACCTCTCTCCCCAGGGGGATCCACCGGAGCTTACCATGACCGAGTACAAGCCCACGGTGCGCCTCGCCACCCGCGACGACGTCCCCAGGGCCGTACGCACCCTCGCCGCCGCGTTCGCCGACTACCCCGCCACGCGCCACACCGTCGATCCGGACCGCCACATCGAGCGGGTCACCGAGCTGCAAGAACTCTTCCTCACGCGCGTCGGGCTCGACATCGGCAAGGTGTGGGTCGCGGACGACGGCGCCGCGGTGGCGGTCTGGACCACGCCGGAGAGCGTCGAAGCGGGGGCGGTGTTCGCCGAGATCGGCCCGCGCATGGCCGAGTTGAGCGGTTCCCGGCTGGCCGCGCAGCAACAGATGGAAGGCCTCCTGGCGCCGCACCGGCCCAAGGAGCCCGCGTGGTTCCTGGCCACCGTCGGCGTCTCGCCCGACCACCAGGGCAAGGGTCTGGGCAGCGCCGTCGTGCTCCCCGGAGTGGAGGCGGCCGAGCGCGCCGGGGTGCCCGCCTTCCTGGAGACCTCCGCGCCCCGCAACCTCCCCTTCTACGAGCGGCTCGGCTTCACCGTCACCGCCGACGTCGAGGTGCCCGAAGGACCGCGCACCTGGTGCATGACCCGCAAGCCCGGTGCCTGAACCGGTGGCCGCCATGGCCCAACTTGTTTATTGCAGCTTATAATGGTTACAAATAAAGCAATAGCATCACAAATTTCACAAATAAATCTAGATTATGGGAGAACTGGAGCCTTCAGAGGGTAAAATTAAGCACAGTGGAAGAATTTCATTCTGTTCTCAGTTTTCCTGGATTATGCCTGGCACCATTAAAGAAAATATCATCTTTGGTGTTTCCTATGATGAATATAGATACAGAAGCGTCATCAAAGCATGCCAACTAGAAGAGGTAAGAAACTATGTGAAAACTTTTTGATTATGCATATGAACCCTTCACACTACCCAAATTATATATTTGGCTCCATATTCAATCGGTTAGTCTACATATATTTATGTTTCCTCTATGGGTAAGCTACTGTGAATGGATCAATTAATAAAACACATGACCTATGCTTTAAGAAGCTTGCAAACACATGAAATAAATGCAATTTATTTTTTAAATAATGGGTTCATTTGATCACAATAAATGCATTTTATGAAATGGTGAGAATTTTGTTCACTCATTAGTGAGACAAACGTCCTCAATGGTTATTTATATGGCATGCATATAAGTGATATGTGGTATCTTTTTAAAAGATACCACAAAATATGCATCTTTAAAAATATACTCCAAAAATTATTAAGATTATTTTAATAATTTTAATAATACTATAGCCTAATGGAATGAGCATTGATCTGCCAGCAGAGAATTAGAGGGGTAAAATTGTGAAGATATTGTATCCCTGGCTTTGAACAAATACCATATAACTTCTAGTGACTGCAATTCTTTGATGCAGAGGCAAAATGAAGATGATGTCATTACTCATTTCACAACAATATTGGAGAATGAGCTAATTATCTGAAAATTACATGAAGTATTCCAAGAGAAACCAGTATATGGATCTTGTGCA
