# Supplementary material for: Targeted Integration of a Super-Exon into the CFTR Locus Leads to Functional Correction of a Cystic Fibrosis Cell Line Model
Source: PLoS One. 2016 Aug 15;11(8):e0161072. doi: 10.1371/journal.pone.0161072 (PMC4985144; doi:10.1371/journal.pone.0161072)
Supplement: S1 Table — (DOCX) [file pone.0161072.s004.docx]

**S1 Table: Primers used for T7EI assay, genotyping and expression analysis**

| **purpose** | **Primer ID** | **sequence 5**’**🡪 3**’ | **product size** |
| --- | --- | --- | --- |
| T7EI assay | T1 | AGAAAGGCTCATGGGAACAAT | 728 bp |
|  | T2 | TGGGTAGTGTGAAGGGTTCAT |  |
| donor detection | P1 | ACGGCCCTATTCTATAGTGTCACC | 376 bp |
|  | P2 | GACGTGAAGAATGTGCGAGA |  |
| 5’ junction | P3 | CCTCCTTTCCTTCTACTCAGTTTTAGT | 876 bp |
|  | P4 | GCCCATGATCACCATCAGCAGACT |  |
| 3’ junction | P5 | AGCTGCAAGAACTCTTCCTCAC | 1528 bp |
|  | P6 | TCATAGTAACATATTCCCTGCCCTA |  |
| *per se* CFTR expression | P7 | GTGATGGAGAATGTAACAGCCTTCTGGG | 470 bp |
|  | P8 | CCACTCAGTGTGATTCCACCTTCTCC |  |
| donor derived CFTR expression | P4 | GCCCATGATCACCATCAGCAGACT | 257 bp |
|  | P7 | GTGATGGAGAATGTAACAGCCTTCTGGG |  |
| Bisulfite sequencing | B1 | GAAATTAATA AAGTTTGGTTTTTTTT | 360 bp |
|  | B2 | CCTCTCTTTAAATCCAATTAACAAC |  |
| Random integration | R1 | ataataccgcgccacatagc (AmpR) | 1339 bp |
|  | R2 | GCCCATGATCACCATCAGCAGACT |  |
